# Sex Chromosomes and Karyotype of the (Nearly) Mythical Creature, the Gila Monster, *Heloderma suspectum* (Squamata: Helodermatidae)

**DOI:** 10.1371/journal.pone.0104716

**Published:** 2014-08-13

**Authors:** Martina Johnson Pokorná, Michail Rovatsos, Lukáš Kratochvíl

**Affiliations:** 1 Department of Ecology, Faculty of Science, Charles University in Prague, Praha, Czech Republic; 2 Institute of Animal Physiology and Genetics, Academy of the Sciences of the Czech Republic, Liběchov, Czech Republic; Virginia Tech, United States of America

## Abstract

A wide variety of sex determination systems exist among squamate reptiles. They can therefore serve as an important model for studies of evolutionary transitions among particular sex determination systems. However, we still have only a limited knowledge of sex determination in certain important lineages of squamates. In this respect, one of the most understudied groups is the family Helodermatidae (Anguimorpha) encompassing the only two venomous species of lizards which are potentially lethal to human beings. We uncovered homomorphic ZZ/ZW sex chromosomes in the Gila monster (*Heloderma suspectum*) with a highly heterochromatic W chromosome. The sex chromosomes are morphologically similar to the ZZ/ZW sex chromosomes of monitor lizards (Varanidae). If the sex chromosomes of helodermatids and varanids are homologous, female heterogamety may be ancestral for the whole Anguimorpha group. Moreover, we found that the karyotype of the Gila monster consists of 2n = 36 chromosomes (14 larger metacentric chromosomes and 22 acrocentric microchromosomes). 2n = 36 is the widely distributed chromosomal number among squamates. In his pioneering works representing the only previous cytogenetic examination of the family Helodermatidae, Matthey reported the karyotype as 2n = 38 and suggested a different chromosomal morphology for this species. We believe that this was probably erroneously. We also discovered a strong accumulation of telomeric sequences on several pairs of microchromosomes in the Gila monster, which is a trait documented relatively rarely in vertebrates. These new data fill an important gap in our understanding of the sex determination and karyotype evolution of squamates.

## Introduction

Squamate reptiles possess a notable variability in sex determination. In this group, sex-determining mechanisms range from environmental sex determination, where the sex of an individual is decided by the environmental conditions present during the sensitive embryonic period and where there are no differences in genotypes between sexes, to genotypic sex determination with highly differentiated heteromorphic sex chromosomes [1], [2]. However, critical evaluation of the data and subsequent phylogenetic analyses indicate that the variability is not distributed equally across major squamate lineages [3]. As far as known, well-supported variability can be found in acrodont lizards [4] and geckos [5]–[7]. On the other hand, emerging molecular-cytogenetic data demonstrate that lineages such as colubroid snakes [8], [9] or iguanas [10]–[12] have remarkably conserved sex chromosomes. However, even basic information is scarce or even totally lacking for many important groups of squamates [2], [3], [13]. This precludes the more reliable phylogenetic analyses of sex determination in this group necessary for understanding the evolution of sex determination. Previously unknown sex chromosomes were recently revealed in several lineages [6], [7], [12], [14]. However, in several popular or phylogenetically important lineages differential staining or methods of molecular cytogenetics often necessary for detection of sex chromosomes have not yet been applied.

One of such neglected lineages is the family Helodermatidae (Anguimorpha) encompassing two extant species, the Gila monster, *H. suspectum*, and the beaded lizard, *H. horridum*, the only lizards with grooved teeth and venom potent enough to kill human beings. The Gila monster, *H. suspectum* has a geographical distribution in southwest USA and northwest Mexico [15], [16], with a threat status of Near Threaten according to IUCN. Two morphological subspecies of *H. suspectum* are recognized, the Reticulate Gila Monster *H. s. suspectum* and the Banded Gila Monster *H. s. cinctum*, but their validity is under debate, since recent molecular study show little phylogeographic structure among populations of the species [17].

**Figure 1:**
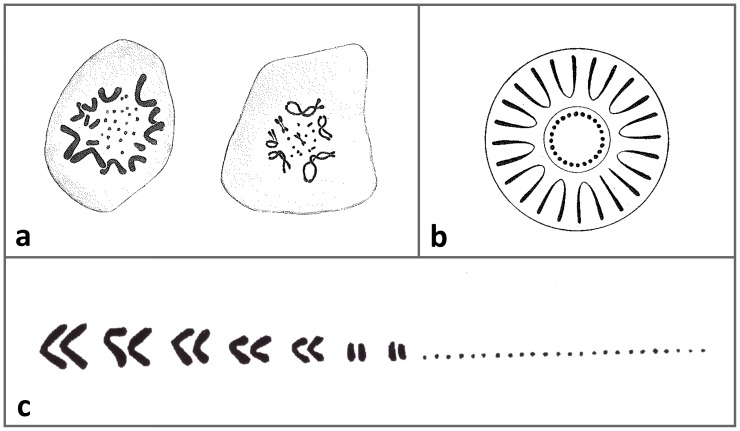
Reprint of the idealized drawings of two examples of male metaphases (a), the schematic depiction of metaphase (b) from Matthey (1931), and the karyotype (c) from Matthey (1933) of the Gila monster.

Although both species of the family are widely studied (for bibliography see [18]; for review of their biology see [16]), as far as we know, only the pioneering seminal articles by Matthey [19], [20] were devoted to the cytogenetics of the family. Matthey [19], [20] described the karyotype of the Gila monster as 2n (diploid chromosomal numbers)  = 38 with 10 metacentric and 4 acrocentric macrochromosomes and 24 dot-like microchromosomes (Fig. 1). He did not report any sexual differences in karyotype. This karyotype is unusual among lizards and would be derived within Anguimorpha, where some species possess a putative ancestral karyotype of 2n = 36 with 12 metacentric macrochromosomes and 24 microchromosomes [21].

To better characterize the karyotype and potentially identify sex chromosomes, we studied the metaphase chromosomes of both sexes of the Gila monster with classical and differential (C-banding) staining as well as by comparative genomic hybridization (CGH) and fluorescent *in situ* hybridization (FISH) with telomeric probes.

## Material and Methods

### Specimen origin

Samples of peripheral blood for chromosomal preparation were obtained from two females and three males of captive bred stock of *H*. *suspectum*, originated from Vail, Tuscon in Arizona, USA. The heparinized blood samples were received courtesy of Dr. Dušan Král and Martina Gregorovičová, the private owners of the animals. Additional tissue samples (muscle) were provided from one preserved male (National Museum in Prague, specimen No. NMP6V-70769) and one preserved female (from the private owner René Urvalský). The procedures were carried out under the supervision and with the approval of the Ethics Committee of the Faculty of Science, Charles University in Prague followed by the Committee for Animal Welfare of the Ministry of Agriculture of the Czech Republic.

### Chromosomal preparations and staining

Metaphase chromosome spreads were prepared from whole blood cell cultures, following the protocol described in Pokorná et al. [6] with slight modifications. Chromosomal preparations were stained with conventional Giemsa solution. C-banding staining was performed as following Sumner [22] with slight modifications as described in our previous article [7]. From each individual at least five Giemsa-stained and five C-banded metaphases were analyzed.

### mtDNA gene "barcoding"

Genomic DNA was isolated from blood or tissue samples from all lizards using the Qiagen DNeasy Blood and Tissue kit (Qiagen Inc., Valencia, CA). The mitochondrial genes ATPase 6, ATPase 8 and COI were amplified through PCR for a molecular characterization of our samples and "barcoding" of the described karyotypes. ATPase 6 and ATPase 8 genes were amplified in a single PCR reaction using the primers L8331 and H9236 [23] and the COI gene was amplified by the universal primers LCO1490 and HCO2198 [24].

PCR reactions were carried out in 50 µl volumes, including 2 µl of each primer (10 pmol/µl), 5 µl of 10×PCR buffer (Bioline), 2.5 µl MgCl 2 (50 mM), 1 µl dNTPs (10 mM each) and 0.5 µl BioTaq DNA polymerase (5 U/µl, Bioline). The PCR cycling conditions for ATPase 6 and ATPase 8 were as follows: 3 min at 94 °C, 35 cycles of 1 min at 94 °C, 45 s at 50 °C and 1 min at 72 °C, followed by a final step of 5 min at 72 °C. The amplification conditions for COI were 94 °C for 3 min, followed by 40 cycles at 94 °C for 30 s, 43–48°C for 30 s and 72 °C for 1 min, with a final step at 72 °C for 5 min. The PCR products were purified and sequenced both directions by Macrogen (Korea). All the obtained sequences were aligned using CLUSTALW [25], included in BioEdit v5.0.9 [26], subsequently analyzed in MEGA v6.0.5 [27], in DnaSP v5.10.1 [28] and subjected to a BLAST search in GenBank for comparison with deposited *Heloderma* sequences. All sequences have been deposited to GenBank under the accession numbers KJ917179-KJ917183.

### Comparative Genomic Hybridization (CGH)

For CGH, genomic DNA was isolated from a muscle of one preserved male and one preserved female. 1 µg of male and 1 µg of female genomic DNA were labeled with biotin-dUTP and digoxigenin-dUTP, respectively, using the Nick Translation Kit (Roche). Afterwards, the two probes were mixed together with 10 µg of sonicated salmon sperm DNA, co-precipitated overnight and resuspended in hybridization buffer (50% formamide, 2×SSC, 10% SDS, 10% dextran sulfate, 1×Denhardt's buffer, pH 7). The *in situ* hybridization of the probe upon the metaphases from both sexes was performed according to our standard protocol [7]. The antibodies avidin-FITC (Vector Laboratories) and anti-digoxigenin-rhodamine (Roche) were applied for the detection of the probe topology upon the metaphase. All slides were counterstained with DAPI and mounted with the anti-fade medium Vectashield (Vector Laboratories).

### *In situ* hybridization with telomeric probes

The pattern of the telomeric repeats within the chromosomes was studied with *in situ* hybridization. The probe for the telomeres was produced and labelled with biotin by a modified PCR protocol, using the primers (TTAGGG)_5_ and (CCCTAA)_5_, without DNA template, based on the methodology of Ijdo et al. [29]. The FISH experiments were performed according to the previously described protocol [7] with three rounds of signal amplification using the avidin-FITC/antiavidin-biotin system (Vector Laboratories). All slides were counterstained with DAPI and mounted with the anti-fade medium Vectashield (Vector Laboratories).

### Image analysis

Images were captured using a Provis AX70 (Olympus) fluorescence microscope, equipped with a DP30BW digital camera (Olympus). The Ikaros karyotyping software (Metasystems) was used for karyotype analysis. DP manager imaging software (Olympus) was used to capture grayscale images and to superimpose the source images with colours to visualize the results of the FISH.

## Results

The PCR amplified successfully all three mitochondrial genes, resulting in sequences of 658 bp for COI, 683 bp for ATPase 6 and 165 bp for ATPase 8, excluding the primer binding sequences. Haplotype analysis of our dataset in DnaSP v.5.10.1 revealed two haplotypes for COI, two haplotypes for ATPase 6 and a single haplotype in ATPase 8. A BLAST search was performed for all our sequences, in order to compare them with sequences deposit in GenBank, derived from the previous publications [17], [30]. The two haplotypes of COI are new, previously not reported sequences, while the haplotypes from ATPase 6 and ATPase 8 have been previously reported [17].

**Figure 2:**
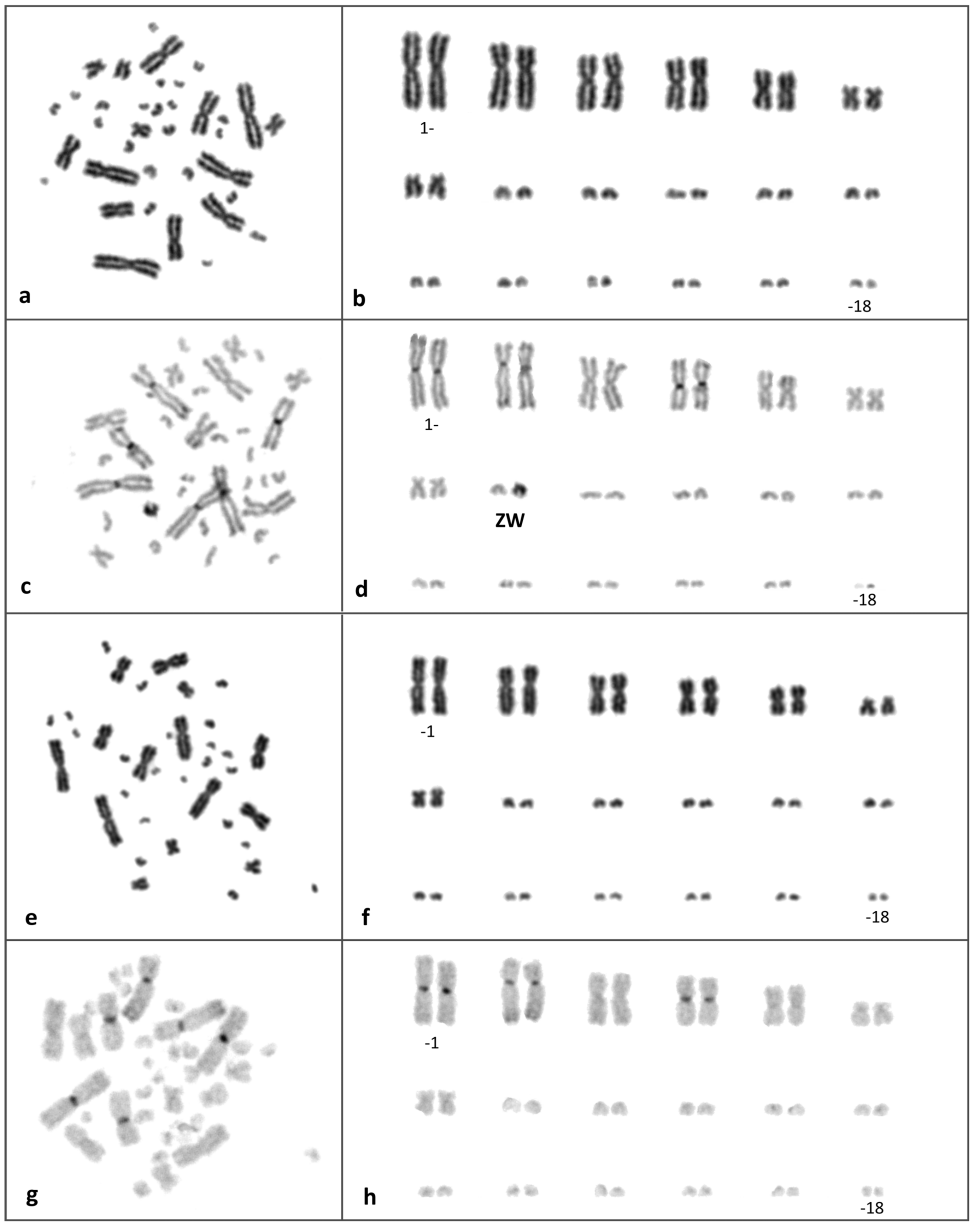
Giemsa (a, b, e, f) and C-banded (c, d, g, h) metaphase chromosomes and karyotypes from a female (a–d) and a male (e–h) of *Heloderma suspectum*. The sex chromosomes are labelled in the C-banded female karyotype (d).

The cytogenetic analyses revealed the same karyotype in all five cytogenetically studied individuals with 2n = 36, consisting of 7 pairs of large to medium-sized metacentric chromosomes and 11 pairs of small acrocentric chromosomes (Fig. 2b, f). No heteromorphic pair was observed among Giemsa-stained chromosomes. Heterochromatin revealed by C-banding was localized in pericentromeric regions of the first, second and fourth pair of macrochromosomes in both sexes. Furthermore, two C-positive bands were identified in the subtelocentric region of the large arm of the second chromosomal pair and another C-positive band in interstitial position in the large arm of the sixth chromosomal pair. One chromosome from the largest pair of microchromosomes was strongly heterochromatic in all females (Fig. 2c, d), but not in males (Fig. 2g, h), which identifies it as the W chromosome. The Z chromosome could be identified by its morphology as the largest microchromosome, unpaired in females in C-banding (Fig. 2d).

**Figure 3:**
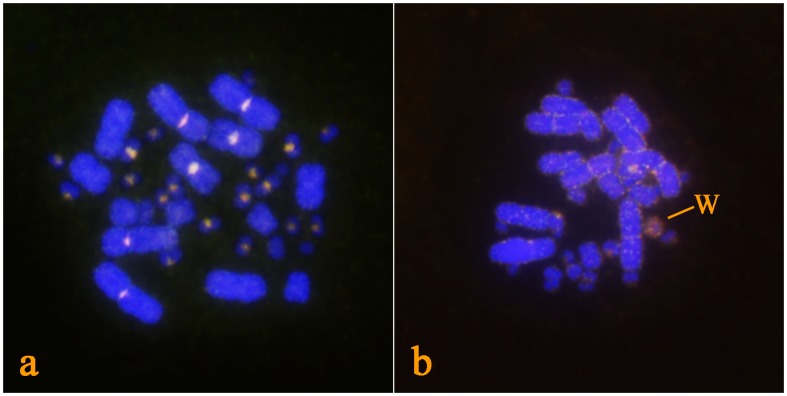
Results of CGH on male (a) and female (b) metaphase of *H. suspectum*. Male genome is stained with FITC (green colour), female genome with rhodamine (red colour). Regions common for genomes of both sexes are yellow (combination of green and red). The W chromosome is indicated on the female metaphase.

**Figure 4:**
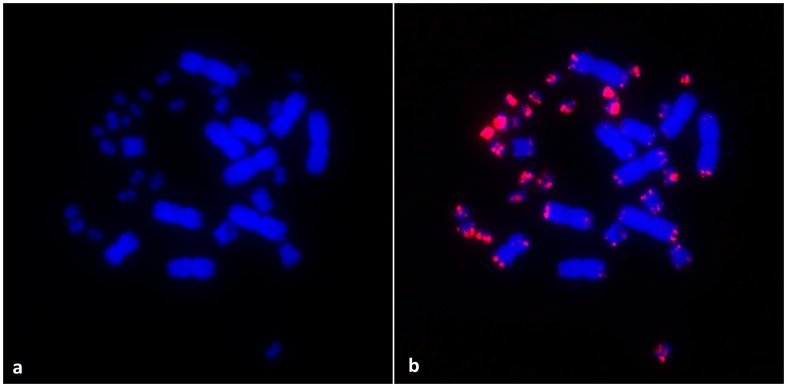
DAPI stained metaphase chromosomes (a) and the results of fluorescent *in situ* hybridization with the telomeric probe (TTAGGG)_n_ (b) in a female of *Heloderma suspectum*.

In CGH, a red stained, middle sized chromosome can be identified in the female, but absent in the male metaphases, showing that sex chromosomes in *H. suspectum* are highly differentiated in sequence content (Fig. 3). The telomeric-specific probes produced typical signals for the telomeres at the ends of all macrochromosomes and several microchromosomes. Strong telomeric signals covering the pericentromeric regions were observed in at least four pairs of microchromosomes (Fig. 4).

## Discussion

We demonstrated that the Gila monster possesses homomorphic ZZ/ZW sex chromosomes with a highly heterochromatic W chromosome (Fig. 2). Female heterogamety is relatively widespread among squamates [3], [31], it has been reported in lacertid lizards and amphisbaenians [32], [33], several lineages of geckos (families Carphodactylidae [7], Phyllodactylidae and Gekkonidae [3], [5]), agamid lizards [4], [14], snakes [8], [9] and monitor lizards [34]. Based on the morphology of sex chromosomes and the phylogenetic distribution of sex-determining systems [3], it seems that female heterogamety evolved independently among most of the among mentioned lineages, however, decisive molecular evidence is largely absent (it has only been proved that sex chromosomes in some agamid lizards and snakes are not homologous [35]).

The sex chromosomes of the Gila monster described here are morphologically similar to sex chromosomes of monitor lizards (Varanidae) [34], the only other family of Anguimorpha with identified sex chromosomes. Taking into account the large conservation of sex determining systems in most squamate lineages [3], the ZZ/ZW sex chromosomes of the Gila monster and monitor lizards could be homologous, but molecular or molecular-cytogenetic studies have to be carried out to test this hypothesis. Considering the phylogenetic relationships as suggested by Pyron et al. [36] (but *cf*. to previous phylogenies suggesting a different topology among families of Anguimorpha [37], [38]), if the sex chromosomes of helodermatids and varanids prove to be homologous, female heterogamety would be likely ancestral for the whole group Anguimorpha.

The karyotype of the Gila monster differs from the previous reports by Matthey [19], [20] even in important basic aspects, i.e. in chromosome number and morphology (*cf*. Figs 1, 2). It is possible that our individuals have a different karyotype than Matthey's due to their different origin and can represent for instance different chromosomal races or subspecies. Unfortunately, Matthey did not refer to the origin of his samples [19], [20]. Our description of the karyotypes of the Gila monsters is accompanied with the sequences of the mtDNA genes serving as a genetic "barcoding". The genetic identity of our karyotyped specimens can be valuable for future comparative research. However, although we greatly value Matthey's pioneering work, in this case we believe that the karyotype was described by him incorrectly. Recent work has revealed little phylogeographic structure of Gila monster populations throughout its range [17]. Moreover, karyotypes in squamates (and in most reptiles, including birds) are generally highly conserved even across large phylogenetic scales [39]–[46] and such intraspecific variation in karyotypes of the Gila monster as suggested between our and Matthey's descriptions seems unlikely, particularly considering the many differences in the reported aspects of chromosomal morphology. Lastly, and most importantly, we have to keep in mind that in the early part of the 20^th^ century microscopy and many cytogenetic techniques were not as developed as they are now. Matthey [19], [20] did not present photographic evidence of the chromosomes, but only idealized drawings of the karyotype (see Fig. 1). At that time, it was not possible to identify the shapes or even the number of smaller chromosomes. For example, even in the human the correct number of chromosomes and their morphology was not reported until 1956 [47].

The chromosomal number 2n = 36 is widely distributed among squamate reptiles [21], [40], [48], [49] and is considered as ancestral for this group [21]. However, this putative ancestral karyotype consists of 12 large metacentric chromosomes and 24 microchromosomes, while the Gila monster has karyotype with 14 biarmed chromosomes varying from large to medium sized and 22 acrocentric microchromosomes. The putative ancestral karyotype was reported in some anguids [21], such as *Ophiodes striatus* [50] or *Diploglossus millepunctatus* [51], while other anguids and monitor lizards have modified karyotypes [21], [34], [41], [52]. Recently, based on physical gene mapping, Srikulnath et al. [52] revealed a high level of synteny of chromosomes among snakes, agamids and monitor lizards and documented several interchromosomal rearrangements among these groups. Similar studies should be carried out to reveal the derived versus plesiomorphic characteristics of the helodermatid karyotype in order to better understand karyotype evolution in Anguimorpha.

Our FISH experiments with telomeric probes showed a large accumulation of telomeric sequences within several euchromatic microchromosomes (Fig. 4). Similar accumulations in microchromosomes have been reported relatively rarely in vertebrates. They are common in avian microchromosomes [53], for example, they were found in the agamid *Leiolepis reevesii* [54] and in the gymnophthalmid *Leposoma osvaldoi* [55]. As far as we know, among fish they were reported only in sturgeons [56]. Nanda et al. [53] speculated that the accumulation of telomeric repeats in microchromosomes can be functional, as they can contribute to the extraordinally high rates of recombination observed in avian microchromosomes. As some other squamates do not possess these accumulations of telomeric sequences in their microchromosomes (e.g. the gecko *Underwoodisaurus milii*; [7]), this group can thus serve as model organisms for comparative studies of the function and evolutionary dynamics of these accumulations. Telomeric-like sequences are sometimes accumulated in the heterochromatic blocks of the sex chromosomes [7], [57], however, it is not the case in the Gila monster. In this species, C-positive bands were observed in non-centromeric regions of the chromosomal pairs 2 and 6 (Fig. 2c, d, g, h). Those bands could be remnants of old centromeric regions after past chromosomal fusions, however, we were not able to support this scenario by the results of the FISH with telomeric probe, as we did not detect any telomeric sequences in these C-positive bands.

In summary, we have described previously unknown sex chromosomes and the chromosomal number and morphology of the Gila monster, a representative of the otherwise highly studied and phylogenetically important lineage of anguimorph lizards. We challenged an 80 year old description of the karyotype of the species, which has been referenced in all subsequent reports of helodermatid cytogenetics. As well as potentially correcting an unfortunate error in past research, our data fills a significant gap in the understanding of sex determination and karyotype evolution in squamates.
